# Retracted: Positive Effects against UV-A Induced Damage and Oxidative Stress on an *In Vitro* Cell Model Using a Hyaluronic Acid Based Formulation Containing Amino Acids, Vitamins, and Minerals

**DOI:** 10.1155/2021/1975827

**Published:** 2021-02-18

**Authors:** BioMed Research International

*BioMed Research International* has retracted the article titled “Positive Effects against UV-A Induced Damage and Oxidative Stress on an *In Vitro* Cell Model Using a Hyaluronic Acid Based Formulation Containing Amino Acids, Vitamins, and Minerals” [1]. As raised on PubPeer [2], there are duplicated figure panels within Figure 2(a):

- The CTR (16 h) and CTR (32 h) panels appear to be identical
- The M-HA (48 h) and M-HA (96 h) appear to be identical
- Skinkò E (48 h) and Skinkò E (96 h) appear to be identical
- Skinkò E (72 h) and CTR (96 h) appear to be identical
- The same cluster of cells can be seen in Skinkò E (48 h), Skinkò E (72 h), and Skinkò E (96 h).

The authors said that the duplications between M-HA (48 h) and M-HA (96 h) and CTR (96) and Skinkò E (72 h) were mistakes introduced during manuscript preparation. A satisfactory explanation was not provided for the remaining concerns and the article is therefore being retracted with the agreement of the editorial board. The authors do not agree to retraction.
